# Co-treatment with disulfiram and glycyrrhizic acid suppresses the inflammatory response of chondrocytes

**DOI:** 10.1186/s13018-021-02262-3

**Published:** 2021-02-12

**Authors:** Chao Li, Li Li, Tian Lan

**Affiliations:** 1 The Sports Medicine of The First Hospital of Kunming, Kunming, 650000 Yunnan China; 2 The Orthopedics Department of Kunming Municipal Hospital of Traditional Chinese Medicine, Kunming, 650000 Yunnan China; 3 The Orthopedics Department of The First Hospital of Kunming, Kunming, 650000 Yunnan China

**Keywords:** Disulfiram, Glycyrrhizic acid, Inflammatory response, Chondrocytes

## Abstract

**Background:**

Osteoarthritis (OA) is a kind of systemic musculoskeletal disorder and a most important factor for causing disability and physical painfulness. Nevertheless, due to the fact that OA can be triggered by multiple etiological factors, this disease is hard to be cured. Therefore, it is of great necessity for us to find novel targets or drugs for OA treatment.

**Materials and methods:**

The chondrocytes were treated with lipopolysaccharide (LPS) and adenosine triphosphate (ATP) to induce pyroptosis in OA. The cell proliferation was detected by Cell Counting Kit-8 assay (CCK-8 assay). Enzyme-linked immunosorbent assay (ELISA) was used for the detection of pyroptosis-related inflammatory factors. Then, the antagonists for gasdermin D (GSDMD) (disulfiram) and high mobility group box 1 (HMGB1) (glycyrrhizic acid) were used to treat the cell model to observe the effects of disulfiram and glycyrrhizic acid on the proliferation of chondrocytes in OA. The protein levels of pyroptosis-related inflammatory factors were measured by western blot, and the levels of aldehyde dehydrogenase (ALDH) and reactive oxygen species (ROS) were measured by corresponding commercial kits.

**Results:**

After chondrocytes were induced by LPS and ATP, the cell proliferation was decreased and the expressions of pyroptosis-related inflammatory factors were increased. Disulfiram and glycyrrhizic acid treatment led to enhanced cell proliferation and increased expressions of pyroptosis-related inflammatory factors, while disulfiram showed better alleviative effects on the inflammation in chondrocytes in OA. However, co-treatment with disulfiram at a high concentration and glycyrrhizic acid did not result in higher proliferation of chondrocytes and alleviated inflammation, but led to oxidative stress.

**Conclusion:**

In conclusion, co-treatment with disulfiram and glycyrrhizic acid at a standard concentration suppresses the inflammatory response of chondrocytes, which may provide guidance for the use of the drugs in the treatment of OA.

## Introduction

Osteoarthritis (OA) is a kind of systemic musculoskeletal disorder and a most important factor for causing disability and physical painfulness [1]. It is the most prevalent chronic joint disease that happens mainly in the elderly over 65, and recent studies have confirmed this disease as an immunopathological disease based on the spectrum between normal control and rheumatoid arthritis [2, 3]. Approximately 300 million adults have been diagnosed with OA according to a report [4]. Patients with this disease have to spend a fortune every year hoping to recover from this disease. Nevertheless, due to the fact that OA can be triggered by multiple etiological factors including aging, obesity, and a genetic predisposition, this disease is hard to be cured [5, 6]. Therefore, it is of great necessity for us to find novel targets or drugs for OA treatment.

In the progression of OA, the pyroptotic levels of chondrocytes and synoviocytes will be elevated [7]. With a recognized N-terminal (NT) domain of ~ 30 kD and a C-terminal (CT) domain of ~ 26 kD, gasdermin D (GSDMD) is an important executor in pyroptosis, and this common element is critical to the activation of different types of inflammasomes [8]. The activation of caspase-11 induced by LPS can trigger robust GSDMD cleavage. GSDMD cleavage can free the N-terminal domain of GSDMD for the formation of oligomerization and the insertion of this domain into the plasma membrane, finally bringing about pore formation. It is generally recognized that pore formation leads to loss of osmotic homeostasis, swelling of the cell, and cell death [9–11].

High-mobility group box 1 (HMGB1) is a nuclear protein that can trigger inflammatory responses when it is activated extracellularly [12]. This highly conserved protein plays its role both inside and outside the cells [13]. Tissue injury and organ dysfunction, which can further induce different kinds of diseases and infection, occur as a result of excessive amounts of extracellular HMGB1 [13, 14]. Recent reports have indicated the crucial role of HMGB1 in inflammatory disorders [13, 15–17]; therefore, antagonists that targeted extracellular HMGB1 have become an ideal therapy for the treatment of inflammatory diseases.

There is no direct research on the role of GSDMD and HMGB1 in chondrocytes of OA, and thus this study aims to use the antagonists for GSDMD (disulfiram) and HMGB1 (glycyrrhizic acid) to treat the model of chondrocytes under the condition of pyroptosis, thereby investigating the synergistic effects and underlying mechanism of these two drugs on OA.

## Materials and methods

### Cell culture and establishment of OA model

C28/I2 chondrocytes were obtained from Otwo Biotech (Shenzhen) Inc. (China). All cells were cultured in Dulbecco’s modified Eagle’s medium (DMEM) (Thermo Fisher Scientific, Inc.) supplemented with 10% fetal bovine serum, 100 IU penicillin, and 100 mg/ml streptomycin (Gibco; Thermo Fisher Scientific, Inc.) at 37 °C in a humidified 95% air and 5% CO_2_ atmosphere.

Lipopolysaccharide (LPS) (1 μg/ml; Sigma) was then used to induce C28/I2 chondrocytes in Dulbecco’s modified Eagle’s medium (DMEM) for 4 h to stimulate the inflammatory responses, and the cells were further treated with adenosine triphosphate (ATP) (3 mM; Sigma) for 1 h to activate the NLR family pyrin domain containing 1 (NLRP1) and NLR family pyrin domain containing 3 (NLRP3) inflammasomes. The C28/I2 chondrocytes in DMEM with the same volume of saline were considered as the control group; the C28/I2 chondrocytes with LPS and ATP treatment were considered as the model group; the C28/I2 chondrocytes added with disulfiram (10 μM; Sigma) based on LPS and ATP treatment were considered as the Disul group; the C28/I2 chondrocytes added with glycyrrhizic acid (10 μM; Sigma) based on LPS and ATP treatment were considered as the GA group; the C28/I2 chondrocytes added with both disulfiram and glycyrrhizic acid based on LPS and ATP treatment were considered as the Disul+GA group. Low concentration of disulfiram was considered as Disul-L (10 μM) and high concentration of disulfiram was considered as Disul-H (20 μM).

### Cell Counting Kit-8 assay (CCK-8 assay)

The C28/I2 chondrocytes were digested and inoculated in the 96-well plate at the concentration of 7000 cells/well to be treated correspondingly for 0 h, 24 h, and 48 h. Then, 10 μl of CCK-8 reagent (Beyotime Institute of Biotechnology) was added to the cell supernatant, which was incubated for 4 h in an atmosphere of 37 °C. The absorbance was measured at 450 nm with an enzyme marker.

### Enzyme-linked immunosorbent assay (ELISA)

C28/I2 chondrocytes (1 × 10^5^ cells/well) were inoculated in a 6-well plate and processed with indicated treatment for 48 h. Concentrations of Interleukin-1β (IL-1β) in cell culture supernatant were measured by IL-1β enzyme-linked immunosorbent assay kit (R&D Systems, MN, USA). Concentrations of Interleukin-18 (IL-18) and HMGB1 in cell culture supernatant were measured by using IL-18 and HMGB1 enzyme-linked immunosorbent assay kits (Dakewe Biotech, Beijing, China) following the manufacturer’s instructions.

### Western blot

C28/I2 chondrocytes (1 × 10^5^ cells/well) were inoculated in a 6-well plate and processed with indicated treatment for 48 h. C28/I2 chondrocytes were washed three times with phosphate buffer saline (PBS) and lysed in the radioimmunoprecipitation (RIPA) lysis buffer (Vazyme Biotech Co., Ltd.). Then, the bicinchoninic acid (BCA) protein assay kit (Roche, Basel, Switzerland) was used for the quantification of protein levels. The protein samples were electrophoresed in sodium dodecylsulphate polyacrylamide gel electrophoresis (SDS-PAGE) for the separation of protein bands. After the transfer of proteins from SDS-PAGE gel onto polyvinylidene difluoride (PVDF) membranes, the proteins were blocked with 5% non-fat skim milk for 2 h. The membranes were incubated with primary antibodies specifically against caspase-1, cleaved caspase-1, GSDMD-N, NLRP3, and GAPDH (1:1,000; Invitrogen), overnight at 4 °C. On the next day, the membranes were incubated with horseradish peroxidase (HRP)-conjugated second antibody (1:5,000; Invitrogen) for 2 h. The bands were visualized by a chemiluminescence substrate kit (ECL Plus; Perkin Elmer Inc., Covina, CA, USA). The expression of target proteins was quantified by Quantity One software (The Discovery Series) after normalization to GAPDH.

### Evaluation of aldehyde dehydrogenase (ALDH) activity

C28/I2 chondrocytes (1 × 10^5^ cells/well) were inoculated in a 6-well plate and processed with indicated treatment for 48 h. Cultured C28/I2 chondrocytes were trypsinized and washed twice with PBS. Afterward, they were analyzed by Aldefluor™ assay (StemCell Technologies, Canada) for the detection of ALDH activity according to the recommendations provided by the manufacturer.

### Detection of reactive oxygen species (ROS)

C28/I2 chondrocytes (1 × 10^5^ cells/well) were inoculated in a 6-well plate and processed with indicated treatment for 48 h. The intercellular ROS level was evaluated by the 2,7-dichlorodi-hydrofluorescein diacetate (DCFH-DA) kits (Molecular probes, Dalian Meilun Biotechnology Co., LTD, China), and the procedures were guided by the instructions given by the manufacturer.

### Statistical analysis

All the measurement data were presented as mean ± standard deviation (SD). Data were processed by the SPSS 19.0 software. Analysis of variance (ANOVA) followed by post hoc *t* test was used for comparison among multiple groups while Student’s *t* test was used for comparison between two groups. *P* < 0.05 indicated that the difference was statistically significant.

## Results

### LPS and ATP resulted in the decreased cell proliferation and the occurrence of pyroptosis in C28/I2 chondrocytes

**Fig. 1:**
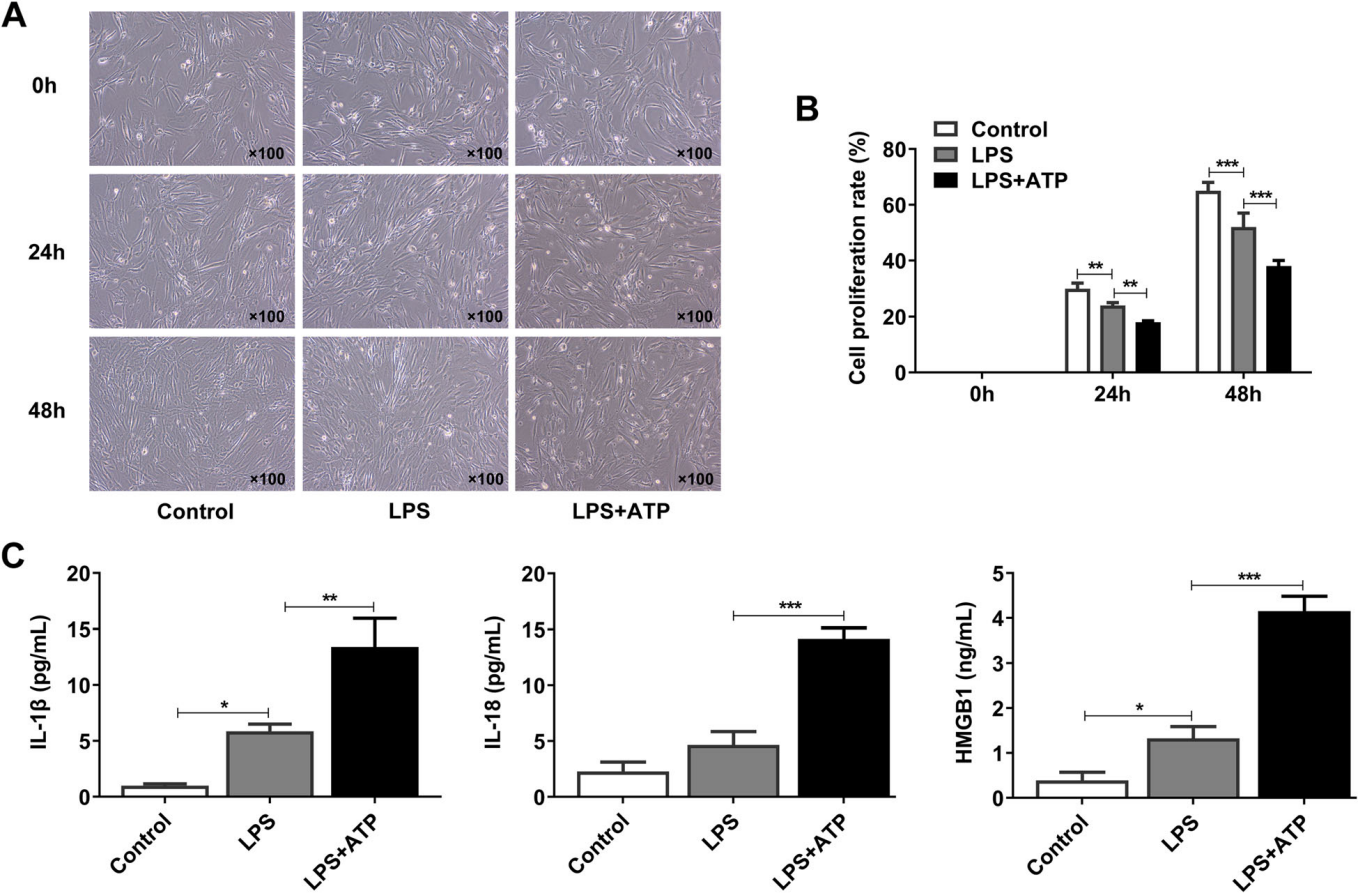
LPS and ATP resulted in the decreased cell proliferation and the occurrence of inflammation in chondrocytes. (**a**-**b**) The cell proliferation of chondrocytes induced by LPS and ATP was detected by CCK-8 assay. (**c**) The levels of pyroptosis-related inflammatory factors were detected by ELISA. *P < 0.05, **P < 0.01, and ***P < 0.001

LPS and ATP were used for the activation of inflammasomes in chondrocytes, which can initiate immune responses in the cells and cell pyroptosis [18]. After the establishment of the in vitro model of OA, the proliferating ability of C28/I2 chondrocytes was detected to confirm the influence of LPS and ATP on cell pyroptosis. As shown in Fig. 1a-b, LPS led to the reduction of cell proliferation rate in C28/I2 chondrocytes by contrast to the control group. However, compared with LPS group, the exposure of C28/I2 chondrocytes to the combined addition of LPS and ATP resulted in even lower rate of cell proliferation in these cells. The activation of inflammasomes can trigger the inflammatory responses, and thus we detected the expressions of pyroptosis-related inflammatory factors, including IL-1β, IL-18, and HMGB1. As exhibited in Fig. 1c, LPS induced the expressions of these pyroptosis-related inflammatory factors, and LPS + ATP group showed higher expressions of these factors than LPS group. Taken together, LPS and ATP combined together resulted in the decreased cell proliferation and the occurrence of pyroptosis in chondrocytes, and LPS + ATP group was thus seen as the model group.

### Co-treatment with disulfiram and glycyrrhizic acid promoted the proliferation and alleviated the pyroptosis in C28/I2 chondrocytes

**Fig. 2:**
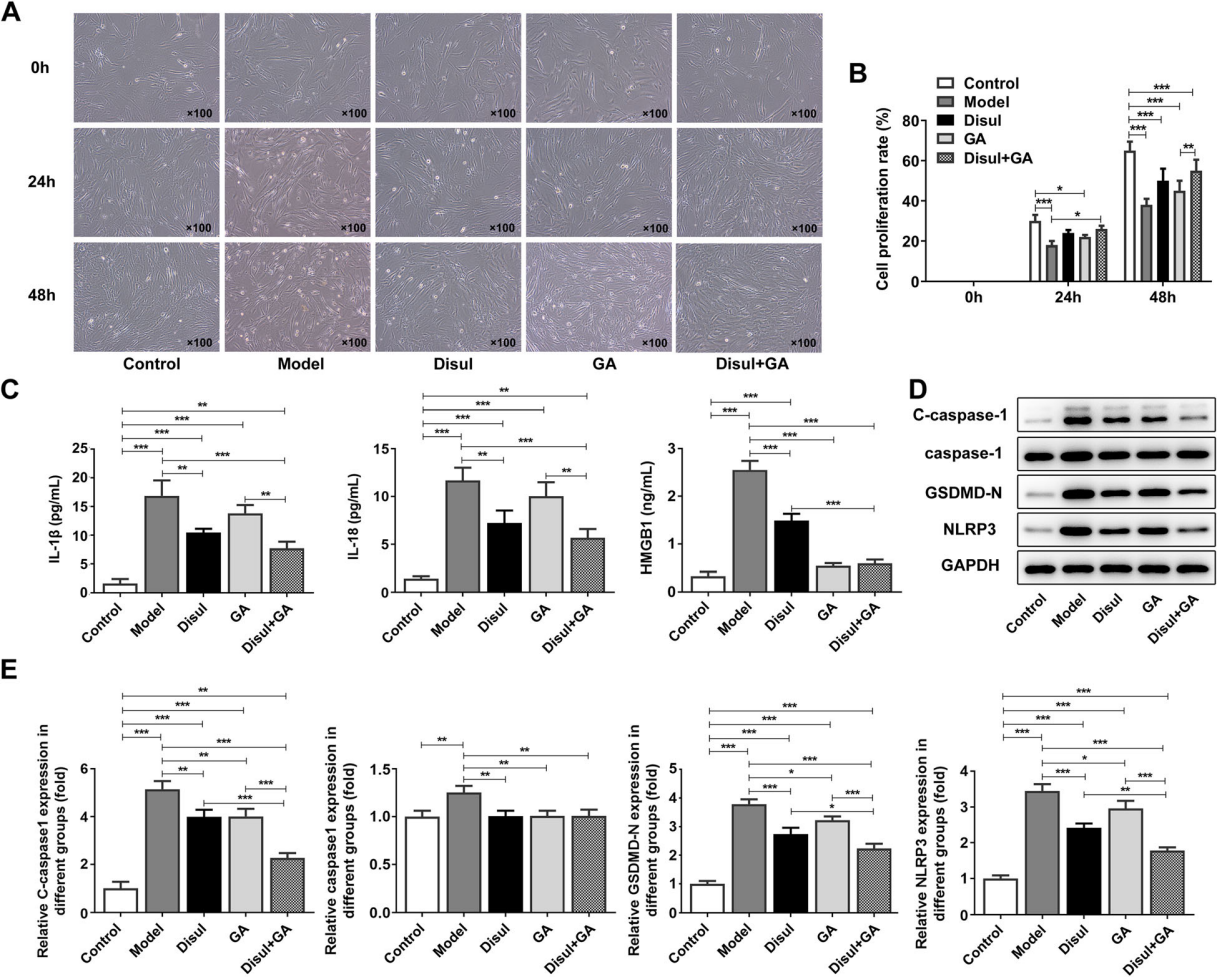
Co-treatment with disulfiram and glycyrrhizic acid promoted the proliferation and alleviated the inflammation in chondrocytes. (**a**-**b**) After co-treatment with disulfiram and glycyrrhizic acid, the cell proliferation of chondrocytes induced by LPS and ATP was detected by CCK-8 assay. (**c**) After co-treatment with disulfiram and glycyrrhizic acid, the levels of pyroptosis-related inflammatory factors were detected by ELISA. (**d**-**e**) After co-treatment with disulfiram and glycyrrhizic acid, the protein and mRNA levels of pyroptosis-related proteins were detected by western blot and PCR, respectively. *P < 0.05, **P < 0.01, and ***P < 0.001

Then, we exposed the chondrocytes to standard doses of disulfiram and glycyrrhizic acid to investigate the effects of disulfiram and glycyrrhizic acid on C28/I2 chondrocytes in OA. Compared with the control group, the model group showed much lower proliferation of C28/I2 chondrocytes. In addition, while treatment of either disulfiram or glycyrrhizic acid separately promoted the proliferation of C28/I2 chondrocytes in comparison to the model group, their co-treatment led to higher proliferation of C28/I2 chondrocytes (Fig. 2a-b). Pyroptosis, together with the secretion of IL-1β and IL-18 that can contribute to inflammation, can cause the occurrence and progression of autoimmune and inflammatory diseases [19]. Thus, the pyroptosis-related inflammatory factors were detected by ELISA assay. The results showed that the dramatically elevated expressions of these factors in chondrocytes induced by LPS and ATP could be reduced by disulfiram and glycyrrhizic acid respectively. In addition, whether the cells were processed by co-treatment with disulfiram and glycyrrhizic acid or the single treatment, expression levels of pyroptosis-related inflammatory factors were all decreased compared with the model group. Co-treatment with disulfiram and glycyrrhizic acid on the cell model could better alleviate pyroptosis than treatment with disulfiram or glycyrrhizic acid alone (Fig. 2c). Consistently, co-treatment with disulfiram and glycyrrhizic acid or single treatment all suppressed the expression of pyroptosis-related genes compared with the model group, and the inhibition effect of co-treatment with disulfiram and glycyrrhizic acid was higher than that of single treatment (Fig. 2d-e). Thus, co-treatment with disulfiram and glycyrrhizic acid promoted the proliferation and alleviated the pyroptosis in chondrocytes.

### High concentration of disulfiram showed marginal effects on the proliferation and pyroptosis in C28/I2 chondrocytes

**Fig. 3:**
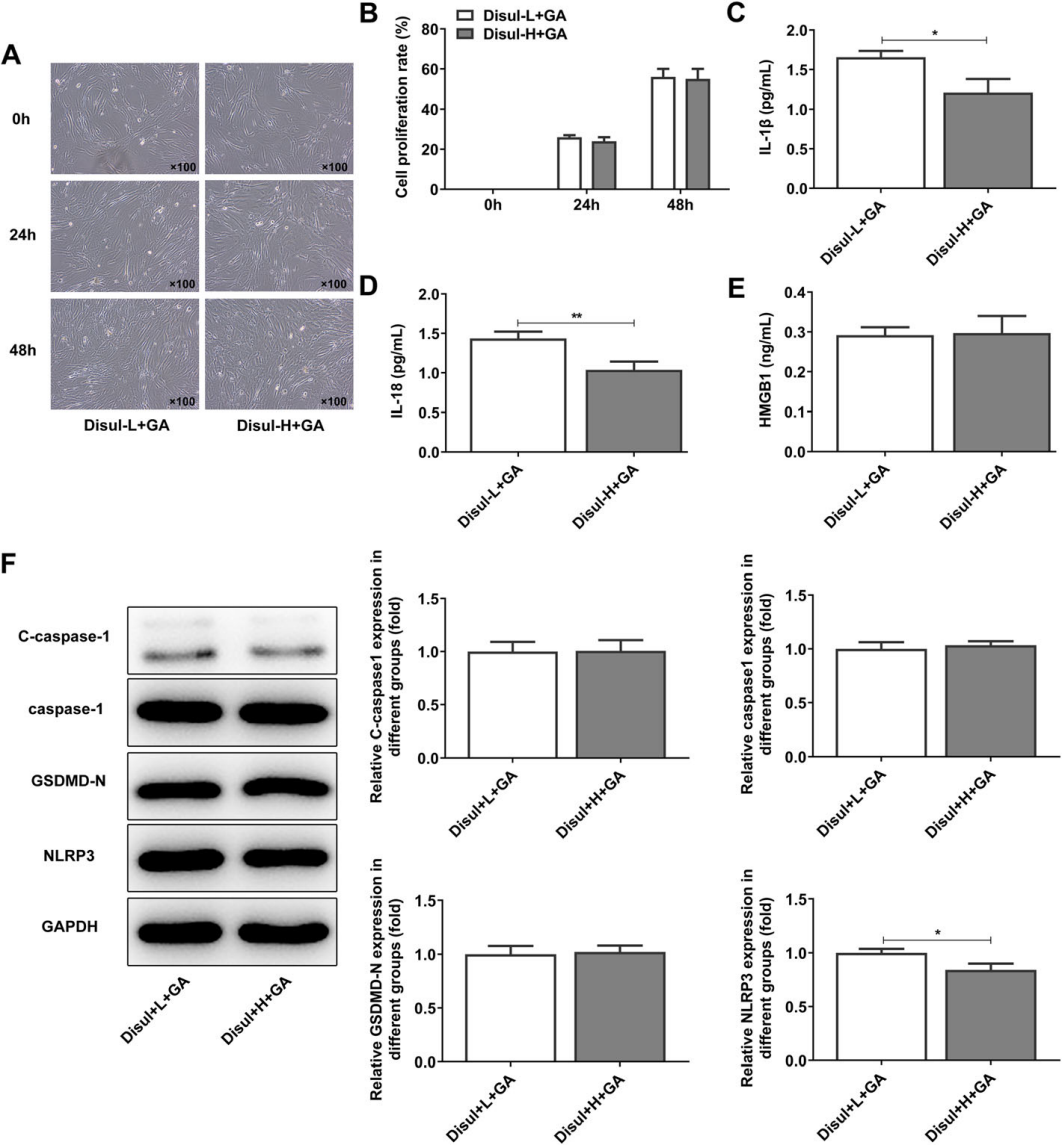
High concentration of disulfiram showed marginal effects on the proliferation and pyroptosis in chondrocytes. (**a**-**b**) After co-treatment with disulfiram at high or low concentration and glycyrrhizic acid, the cell proliferation of chondrocytes induced by LPS and ATP was detected by CCK-8 assay. (**c**-**e**) After co-treatment with disulfiram at high or low concentration and glycyrrhizic acid, the levels of pyroptosis-related inflammatory factors were detected by ELISA. (**f**) After co-treatment with disulfiram at high or low concentration and glycyrrhizic acid, the expressions of pyroptosis-related inflammatory proteins were detected by western blot. *P < 0.05 and **P < 0.01

It was evident from the statistics and results that disulfiram played a better effect on the inhibition of cell inflammation and proliferation than glycyrrhizic acid in chondrocytes. Therefore, we then compared the effects of co-treatment with low concentration of disulfiram (Disul-L) and glycyrrhizic acid, and co-treatment with high concentration of disulfiram (Disul-H) and glycyrrhizic acid on C28/I2 chondrocytes. It was shown in Fig. 3a-b that high concentration of disulfiram did not lead to obvious reduction in the proliferation of chondrocytes, compared with low concentration of disulfiram. The expressions of IL-1β and IL-18 were decreased after co-treatment with high concentration of disulfiram and glycyrrhizic acid in chondrocytes (Fig. 3c-d). However, the expression of HMGB1 showed almost no change whether the concentration of disulfiram added in chondrocytes was high or low (Fig. 3e). The levels of pyroptosis-related proteins also showed no obvious change except for decreased NLRP3 expression after high concentration of disulfiram was added (Fig. 3f). From the results above, we could confirm that high concentration of disulfiram showed marginal effects on the proliferation and pyroptosis in chondrocytes.

### High concentration of disulfiram given in C28/I2 chondrocytes resulted in oxidative stress

**Fig. 4:**
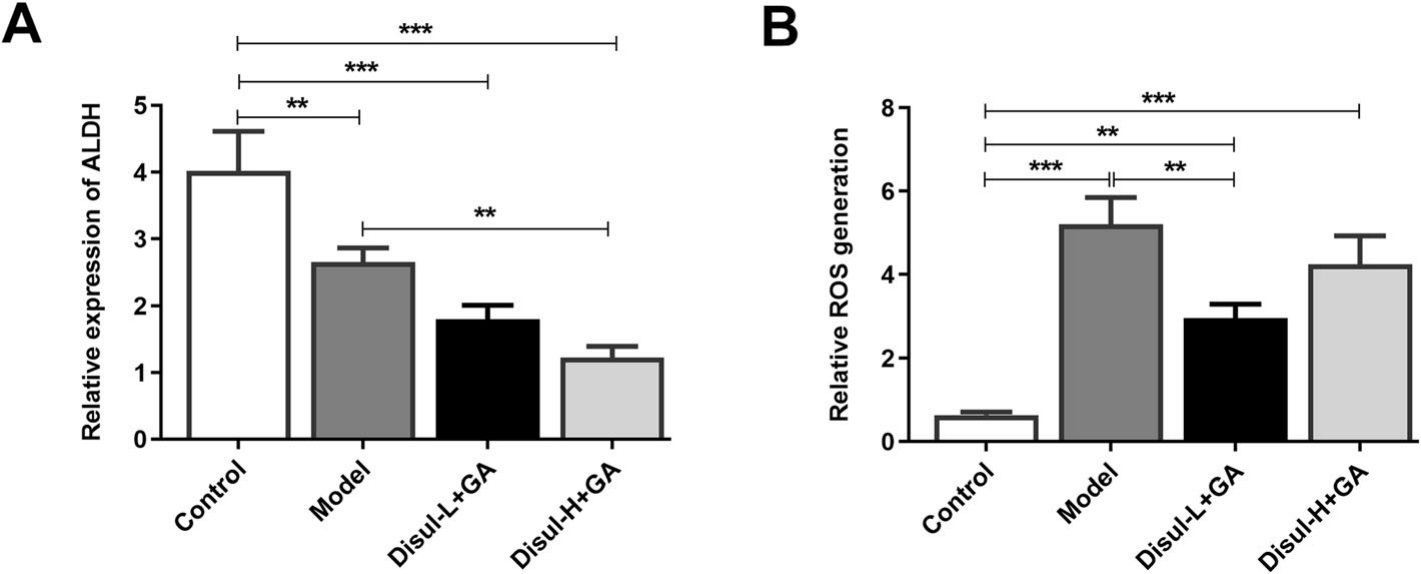
High concentration of disulfiram given in chondrocytes resulted in oxidative stress. The expressions of (**a**) ALDH and (**b**) ROS were respectively measured by corresponding commercial kits. **P < 0.01 and ***P < 0.001

Disulfiram is also the inhibitor of ALDH, and thus the level of ALDH was detected. Model group exhibited lower level of ALDH than the control group, and co-treatment of disulfiram in low or high concentration and glycyrrhizic acid decreased the level of ALDH (Fig. 4a). However, Disul-H + GA group showed much lower level of ALDH than Disul-L + GA group. ROS has been seen as a major force in the process of pyroptosis. Previous studies have shown that pyroptotic cell death is related to irreversible mitochondrial damage and ROS accumulation [20]. The level of ROS in chondrocytes induced by LPS and ATP was alleviated by disulfiram in low or high concentration and glycyrrhizic acid, but high concentration of disulfiram resulted in the aggravation of ROS products (Fig. 4b). Thus, high concentration of disulfiram resulted in oxidative stress.

## Discussion

OA is the most prevalent joint disease with high incidence among older people [2]. It is estimated that over one-third of people in the USA who are over 60 are living with pain arising from OA [21, 22]. Current therapies for OA are acupuncture, weight loss, balance exercises, yoga, cognitive behavioral therapy, kinesiotaping, thermal modalities, radiofrequency ablation, topical NSAIDs, intraarticular steroid injections, and chondroitin sulfate, etc. [23]. However, these treatment methods can only mitigate the pain brought by OA but cannot achieve ideal therapeutic effects, possibly owing to the relatively poor understanding of the specific molecular mechanism regarding the pathogenesis of OA, which is incurred by multiple factors.

Inflammation plays an important role in osteoarthritis [24]. Excessive inflammatory response in the host will cause damage to the cells and tissues and lead to inevitable cell death [25]. During this process, pyroptosis will occur as a vital form of programmed cell death. Pyroptosis is an inflammatory cell death mode which is characterized by pore formation on the plasma membrane, cell swelling, and plasma membrane disruption [26, 27]. Its underlying mechanism involves the activation of caspase-1 and its related inflammasomes such as caspase-11, which lead to host defense against bacterial infections [19]. The levels of pyroptosis in chondrocytes and fibroblast-like synoviocytes were increased in OA, and a large amount of IL-1β could be found in chondrocytes in OA [7]. Thus, in this study, LPS and ATP are used for inflammasome activation, which can further induce cell pyroptosis of chondrocytes so as to construct the cell model of OA [18]. After inflammasome activation, the levels of pyroptosis-related inflammatory factors such as IL-1β were dramatically elevated. Consistently, a study has indicated that pyroptosis was involved in IL-1β secretion ex vivo [28].

GSDMD is a candidate for the pyroptotic pore formation [29]. Recent research has confirmed that GSDMD was a downstream effector of caspase-1 or caspase-11 with the ability to regulate pyroptosis and release IL-1β to extracellular space [10]. Also, strong evidence has showed that activation of caspase-1 proteolytically matures pro-IL-1β and pro-IL-18 and therefore induces pyroptosis partially through cleavage of gasdermin D (GSDMD) [7]. Research has suggested that inhibition of GSDMD could alleviate inflammasome-induced pyroptosis [30]. One study that used mouse model for investigation also mentioned that deletion of GSDMD in the kidney tissues of mice alleviated the liver injury in mice, suggesting the contribution of GSDMD to the development of liver injury [31]. Without stimulation, GSDMD with full length can be intact with the N-terminal (GSDMD-N) and C-terminal (GSDMD-C) regions that interact with each other [32]. The N-GSDMD fragments can be generated when caspase-1 is used for cleavage of inflammasome-activated macrophages, and the subsequent pore formation of oligomerized N-GSDMD can lead to pyroptosis and the facilitation of IL-1β release [33]. HMGB1 has been considered as an important protein related to the inflammatory diseases as it can regulate inflammation and infectious injury [32]. Recent reports have demonstrated the important role of HMGB1 in the activation of caspase-1 and its contribution to the inflammatory reaction in liver ischemic injury [34]. Disulfiram is a cheap and therapeutically effective drug used for alcohol dependence and cancer treatment [35]. Disulfiram and its metabolites formed in vivo were recognized as antibacterial agents against thirty species of Gram-positive and Gram-negative bacteria [36]. Together with glycyrrhizic acid, there is no direct evidence that shows its role in mediating inflammatory diseases. In the present study, the combined effect of disulfiram and glycyrrhizic acid in OA were studied. Herein, disulfiram and glycyrrhizic acid both increased the proliferation and alleviated the inflammation of chondrocytes, while disulfiram showed better effects in the increase of proliferation rate and the alleviation of inflammation. Thus, disulfiram in high concentration was used together with glycyrrhizic acid on chondrocytes in OA. However, high concentration of disulfiram showed marginal effects on the proliferation of chondrocytes and resulted in oxidative stress.

In conclusion, co-treatement with disulfiram and glycyrrhizic acid in a standard concentration suppresses the inflammatory response of chondrocytes, which may provide additional guidance for the use of the drugs in the treatment of OA.

## Data Availability

The datasets used and/or analyzed during the current study are available from the corresponding author on reasonable request.
